# Tonsillar Lymphoma in Children According to Age Group: A Systematic Review

**Published:** 2018-03

**Authors:** Guilherme -Machado de Carvalho, Henrique- Furlan Pauna, Agrício- Nubiato Crespo, Reinaldo- Jordão Gusmão, Alexandre- Caixeta Guimarães

**Affiliations:** 1 *Department of Otorhinolaryngology, Head and Neck Surgery,* * University of Campinas* *, Campinas, * *São Paulo, Brazil.*

**Keywords:** Children, Lymphoma, Tonsillar neoplasm, Lymphadenopathy, Tonsillar asymmetry

## Abstract

**Introduction:**

Lymphoma is a common malignant tumor of the head and neck occurring during childhood. Early diagnosis is very important in terms of prognosis in patients with tonsillar lymphoma.Our objective was to evaluate the clinical manifestations of pediatric tonsillar lymphoma according to different age groups.

**Materials and Methods:**

A systematic review of available English, Spanish, or Portuguese literature from January 1996 to June 2012 was performedin the BIREME, Cochrane, IBECS, Lilacs, PubMed/Medline, SCIELO, and Scopus databases, using “tonsillar lymphoma” and “children” as keywords. Inclusion criteria were pediatric case reports, patients aged up to 18 years, and information on clinical features at the time of diagnosis.

**Results:**

Out of 87 identified publications, 13 articles were selected describing 53 patients. Tonsillar asymmetry was the most common sign. Snoring is a common sign in patients aged under 5 years; clinical lymphadenopathy is frequent among patients aged between 6 and 10 years; and dysphagia is a common sign in patients between 11 and 18 years of age. Burkitt’s lymphoma is the most common form among all ages studied, followed by B-cell lymphoma.

**Conclusion:**

Clinical manifestations differ according to age group. However, tonsillar asymmetry is the most frequent sign regardless of age group.

## Introduction

Lymphoma is a frequent malignant tumor of the head among pediatric patients, and chronic lymphadenopathy of the neck is one of the main clinical signs. Among the types of lymphoma, non-Hodgkin lymphoma has the highest incidence in this pediatric population (1–4). Up to 20% of patients have extra-nodal involvement, with the most frequent site being the palatine tonsil (3,5,6). Some studies have noted the involvement of Waldeyer’s ring in 15% of pediatric cases of lymphoma (5,7,8). According to the Ann Arbor Staging System (9), the initial stage and the size of the tonsillar lymphoma at diagnosis are related to survival rate free of disease (4,5,9,10).

Asymmetry of the palatine tonsils also seems to be related to tonsillar lymphoma. Recently, a meta-analysis and systematic review showed an association between asymmetry of the palatine tonsils and tonsillar lymphoma (8,10). These studies also demonstrated that tonsillar asymmetry, changes in the appearance of the tonsil, and lymphadenopathy are common in the pediatric population with tonsillar lymphoma (8,10).

Early diagnosis and treatment are both important in terms of the prognosis of tonsillar lymphoma. Therefore, knowledge of the most frequent clinical features of pediatric lymphoma according to each age group is a useful tool for identifying patients with suspected tonsillar lymphoma (1,5,10). Although children can present different clinical manifestations of the disease with age, no previous studies or systematic reviews have compared clinical manifestations of tonsillar lymphoma in children according to age groups. Therefore, this study aims to describe the main clinical manifestations of tonsillar lymphoma and correlate with each age group by means of a systematic review.

## Materials and Methods

This systematic review of the literature searched for studies on BIREME, Cochrane, IBECS, Lilacs, PubMed/Medline, SCIELO, and Scopus. The studies were in English, Portuguese, or Spanish and reported on lymphoma of the tonsils in children. No regional limitations were applied and children from different countries were included. The studies for this review were dated between January 1996 and June 2012. The year 1996 was randomly selected to simplify the search and because there were just a few articles before that year, and many of them were not available in the internet. Duplicates were excluded manually. The MeSH terms and keywords used for this systematic review were “tonsillar lymphoma” and “children”. Two of the authors (GMC and ACG) were responsible for searching and reading all the selected studies. Importantly, this systematic review followed the Preferred Reporting Items for Systematic Reviews and Meta-Analyses (PRISMA) criteria and the recommendations of the Cochrane Collaboration (11).

The inclusion of studies was analyzed by both of the authors. Original reports and case reports were permitted, and included patients aged up to 18 years and a clinical description of the palatine tonsils at the time of the diagnosis. In addition, all included studies had a histopathological analysis confirming a diagnosis of lymphoma. However, since tonsillar lymphoma is a rare condition in the pediatric age group, all case reports were included to increase the sample size and to avoid any potentially important loss of information. Review articles with no description of clinical cases and those with no clear definition for the presence or absence of lymphoma of the palatine tonsils were excluded. Thereby, studies with no correlation of age and clinical features were also excluded. Any clinical features not mentioned in the studies were considered absent during the evaluation of the authors.

We performed a preliminary selection of relevant articles by reading the title and abstracts, and excluded studies as per our inclusion criteria; the remaining papers were then fully read. Both authors independently read the articles and extracted relevant data regarding the review; discrepancies were resolved by mutual consensus.

*Definition of the age groups*

Patients were divided into three groups according to age. Group I included patients up to 5 years of age, Group II included patients aged between 6 and 10 years, and Group III included patients between 11 and 18 years of age. The division into three age groups was performed by the authors when collecting the available data. Therefore, we could observe and correlate clinical features according each age group, searching for a specific or satellite sign that could suggest lymphoma in each of the groups.

*Statistical analysis*

Clinical features among the three groups were compared using the -square test, with p-values smaller than 0.05 considered statistically significant. The statistical analysis was performed using the software Sigma XL version 6.22 (SigmaXL^®^ Inc, Canada).

*Ethics*

This study was approved by our Institution’s Ethical Committee.

## Results

**Fig 1:**
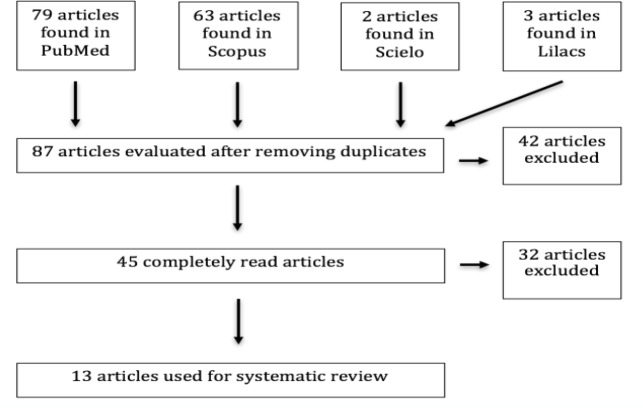
Flowchart of the results in the search of lymphoma of the tonsils in children.

In total, 147articles were found relating to lymphoma of the tonsils in children, from which, 60 duplicates were excluded. Of the 87 remaining studies, 42 were excluded because of a lack of clinical data (many focused on pathology, immunology, or gene expression). In addition, two articles were excluded because they were literature reviews, with no description of clinical cases. Two further studies were excluded since they were reports of cerebellar tonsils diseases. Of the 45 articles read in full, five were excluded because they provided no detailed information regarding their case reports; six were excluded because the age of the subjects was not reported; 17 were not case reports of lymphoma of the tonsils or asymmetric palatine tonsils; three studies were excluded for being reviews of the literature without case reports; and one editorial was excluded. Ultimately, 13 articles remained for systematic review (Fig. 1).

We found 87 published articles using the MeSH terms; however only 13 (15%) were included in this systematic review that matched to our criteria. Overall, we obtained the clinical data for 53 tonsillar lymphoma patients, and analyzed their clinical features (Table.1).

**Table 1:** Summary of the age groups and characteristics of patients included in this systematic review.

Author	Age (years)	Age group	Sex
**Sayed K et al**	17	III	F
**Sayed K et al**	16	III	F
**Sayed K et al**	16	III	M
**Sayed K et al**	15	III	F
**Banthia et al **	14	III	M
**Sayed K et al**	14	III	M
**Dolev Y et al**	13	III	F
**Sayed K et al**	13	III	M
**Sayed K et al**	13	III	M
**Dolev Y et al**	12	III	M
**Sayed K et al**	12	III	F
**Sayed K et al**	12	III	M
**Tewfik TL**	12	III	M
**Dolev Y et al**	10	II	M
**Garcia Ortega**	10	II	M
**Gheoghe et al**	10	II	M
**Tewfik TL**	10	II	M
**Dolev Y et al**	9	II	M
**Sayed K et al**	9	II	M
**Sayed K et al**	9	II	F
**Tewfik TL**	9	II	M
**Garavello et al**	8	II	F
**Sayed K et al**	8	II	M
**Sayed K et al**	8	II	M
**Sayed K et al**	8	II	M
**Dolev Y et al**	7	II	M
**Maitra A et al**	7	II	M
**Sayed K et al**	7	II	M
**Sayed K et al**	7	II	M
**Sayed K et al**	7	II	M
**Sayed K et al**	7	II	M
**Garavello et al **	6	II	M
**Papouliakos et al **	6	II	M
**Smitheringale**	6	II	M
**Smitheringale**	6	II	M
**Carvalho et al**	5	I	F
**Zeglaiou I et al**	5	I	F
**Sayed K et al**	5	I	M
**Sayed K et al**	5	I	M
**Sayed K et al**	5	I	?
**Dolev Y et al**	4	I	F
**Sayed K et al**	4	I	M
**Sayed K et al**	4	I	F
**Sayed K et al**	3	I	M
**Sayed K et al**	3	I	F
**Sayed K et al**	3	I	M
**Sayed K et al**	3	I	M
**Sayed K et al**	2	I	F
**Sayed K et al**	2	I	M
**Smitheringale**	2	I	M
**Smitheringale**	2	I	M
**Williams et al **	2	I	M
**Smitheringale**	1	I	F

F = female, M = male, I = 0 to 5 years; II = 6 to 10 years; III = 11 to 18 years

Eighteen patients were included in Group I (patients up to the age of 5 years), 55.0% of whom were male. Tonsillar asymmetry (72.2%) and snoring (33.3%) were the most frequent manifestations in Group I. Dysphagia (16.7%), fever (11.1%), loss of weight (5.5%), and lymphadenopathy (5.5%) were uncommon features in this group (Table 2).

**Table 2:** Description of the clinical features (%) and p-values with  -square analysis.

	Group I (n=18)	Group II (n=22)	Group III (n=13)	P
**Sex**				
** Male** ** Female**	55.0 45.0	90.9 9.1	61.5 38.5	0.0453^+^
**Lymphadenopathy**	5.5	45.5	0.0	<0.001 ^+^
**Tonsillar asymmetry**	72.2	68.2	73.5	0.5595
**Palatine tonsils mucosa changes**	27.8	22.7	15.4	0.7182
**Dysphagia**	16.7	13.6	46.2	0.0643
**Snoring**	33.3	13.6	15.4	0.2675
**Fever**	11.1	4.5	0.0	0.4
**Weight loss**	5.5	9.1	0.0	0.5312

+ = statistically significant

Burkitt’s lymphoma was the most common type of lymphoma in this age group (55.6%), followed by B-cell lymphoma (38.9%) and T/NK lymphoma (5.6%) (Table.3).

**Table 3:** Description of diagnosed tonsillar lymphoma and its correlation with age and sex**.**

**Lymphoma**	**Patients (n)**	**Mean age (range)**	**Age group**			**Male**	**Female**
			**I** **(n=18)**	**II** **(n=22)**	**III** **(n=13)**		
**Burkitt’s**	31	8.22 (1–17)	10	12	9	23	8
**B-cell**	15	6.93 (2–15)	7	5	3	10	4
**NHL histiocytic**	2	10.0 (7–13)	–	1	1	1	1
**T lymphoblastic**	2	6	–	2	–	2	–
**Hodgkin**	1	10	–	1	–	1	–
**T/NK cell**	1	5	1	–	–	–	1
**Precursor B-cell lymphoblastic**	1	7	–	1	–	1	–

In Group II (patients aged 6 to 10 years), 22 patients were included. Surprisingly, male patients were the clear majority of this group, representing 91% of all cases. Tonsillar asymmetry was found in 68.2% of cases, and clinical lymphadenopathy in 45.5% of cases. Snoring and dysphagia were both described in 13.6% of cases, while weight loss (9.1%) and fever (4.5%) were also identified in this group. Burkitt’s lymphoma was diagnosed in 54.5% of patients, with other types of lymphoma including B-cell lymphoma (22.7%), lymphoblastic lymphoma (9.1%), Hodgkin’s lymphoma (4.5%), histiocytic lymphoma (4.5%), and precursor B-cell lymphoblastic lymphoma (4.5%).

InGroup III (patients aged 11 to 18 years), the majority (61.5%) of the 13 patients were male. Tonsillar asymmetry was present in 73.5% of cases (becoming the most important suspicious factor for this age group), and lymphadenopathy during physical examination was not present in any case. Dysphagia was identified in 46.2% of cases. Symptoms such as weight loss, or fever were not observed in this group. Burkitt’s lymphoma was again the most frequently diagnosed lymphoma, in 69.2% of cases, followed by B-cell lymphoma (23.1%), and one (7.7%) case of histiocytic lymphoma. Changes in the mucosa covering the palatine tonsils (i.e. any disturbance in the appearance of the tonsils) were consistently present in all three groups; however, this was more prevalent in Group I than in the other two groups (27.8%, 22.7%, 15.4% for Group I, Group II, and Group III, respectively). Finally, immunosuppression and radiotherapy had no relevant difference among the three groups. Statistical analysis showed significant differences results for gender distribution (P=0.045) and for lymphadenopathy (P=0.009) between the groups.

## Discussion

Tonsillectomy is the most common surgical procedure performed by an otolaryngologist (12). Enlargement of the palatine tonsils according to Brodsky’s criteria, recurrent infections according to Paradise’s criteria, sleep obstructive apnea, and asymmetrical enlargement of the palatine tonsils (suspicious of malignancy) are common indicators for the procedure (12). Because non-Hodgkin’s lymphoma is the most common malignant disease within the lymphatic Waldeyer’s ring (occurring up to 80% of the time in the palatine tonsils), an otolaryngologist should suspect malignancy in cases of asymmetry of the palatine tonsils associated with other signs and symptoms (3).

Guimarães et al. found that asymmetric tonsils were present in 73.2% cases of lymphoma. They also found a relationshipbetween the asymmetry of the tonsils and lymphoma (a likelihood ratio of 43.5 for children with asymmetric tonsils, and a likelihood ratio of 8938.4 for children with asymmetric tonsils and other suspicious signs of malignancy) (10). In another study, Guimarães et al. observed tonsillar enlargement in 72.7% of children, cervical lymphadenopathy in 30.3%, and the presence of B symptoms in 16% of patients (8). Unlike this study, the present article describes clinical features separately according to different age groups. We found that the majority of cases of male patients were within the group of children aged from 6 to 10 years; also, we found that lymphadenopathy was the most frequent finding within this group, with statistically significant difference between groups (P<0.0001).

Asymmetrical enlargement of the palatine tonsils is considered a dilemma for many otolaryngologists due to the risks of the tonsillectomy itself, such as post-operative bleeding and pain, and also the risk of general anesthesia (13). A systematic review performed by Hwang et al. showed no evidence that would justify tonsillectomy in cases of tonsillar asymmetry without any other associated symptoms in both children and adults. However, this study suggested that the surgical team perform the surgery in cases of widespread lymphadenopathy with rapid increase, or when systemic symptoms are associated (14).

Tumors of the head and neck, especially in pediatric patients, may present in many ways, and generally as a growing painful mass in the neck, or as systemic symptoms such as fever or weight loss. According to the variability and location of the tumor, symptoms such as dysphagia, hoarseness, stridor, involvement of cranial nerves, epistaxis, facial asymmetry, or tearing might occur (2).

Geographical differences should also be highlighted (2). According to the International Classification of Childhood Cancer, approximately 77,111 cases of pediatric cancer in children up to 14 years of age were registered between 1978 and 1997 in Europe only. Among these cases, only 0.9% were diagnosed with lymphoma (1). In the United Kingdom, a double peak of incidence of Hodgkin’s lymphoma and B-cell lymphomawas observed; the first is common in patientsaged 20 to 39 years and the second is more common in patients aged 70 to 79 years (15). Hodgkin’s lymphoma was reported in 4% of children aged up to 14 years, and in 12% of patients aged 15 to 30 years in the United States (9). In Equatorial Africa, approximately 50% of patients with pediatric neoplasms are diagnosed with lymphoma (9).

Our study shows that tonsillar asymmetry is the most common finding across the three age groups, making it an important factor during medical evaluation. However, the distribution of tonsillar asymmetry between the three different age groups is quite similar and must be considered a suspicious sign among all age groups. On the other hand, many studies have found no malignancy in the palatine tonsils after tonsillectomy. For example, Oluwasanmi et al. (2008) presented two patients among 87 that were diagnosed with lymphoma, and Randall et al. (2007) found an incidence of 0.011% of malignancy among 54,901 adult patients with tonsillar asymmetry (14). In 1996, Dohar and Bonillareviewed 2012 tonsillectomies in children due to tonsillar asymmetry, and found only one (0.049%) case of lymphoma. According to Berkowitz and Mahadevan, no cases of lymphoma were foundafter reviewing the charts of 46 pediatric patients with exclusive signs of tonsillar asymmetryin 1999 (12).

According to Papouliakos et al., tonsillar asymmetry must be considered a “high risk” for malignancy in adults with a previous history of neoplasm, firm nodule of the neck, visible lesions within the palatine tonsil, lymphadenopathy, and systemic symptoms. Additionally, Vasilakaki et al. showed a peak of incidence between the sixth and seventh decades, and a slight predominance among male patients. For the pediatric group, the presence of unilateral enlargement of the palatine tonsils, lymphadenopathy of the neck, or systemic symptoms would already be indicative of malignancy (16–18).

Our study reinforces the suspicion of malignancy in cases of asymmetrical enlargement of the palatine tonsils in children, especially when accompanied by other signs and symptoms such as fever, dysphagia, snoring, or cervical lymphadenopathy. A final recommendation for tonsillectomy in this group of patients seems a viable option since therapeutic possibilities and survival rates increase when early diagnosis is performed. 

## Conclusion

We observed that tonsillar asymmetry was the most prevalent symptom of lymphoma in children. Lymphadenopathy is usual in patients aged from 6 to 10 years. Signs such as fever and weight loss had no significant relationship with any group evaluated.

In patients aged up to 5 years, we can consider tonsillar asymmetry and snoring as the most common clinical manifestations, while between the ages of 6 and 10 years, tonsillar asymmetry and lymphadenopathy are more common. Finally, between 11 and 18 years of age, tonsillar asymmetry and dysphagia are the most frequent findings. Burkitt's lymphoma is more common in all groups, followed by B-cell lymphoma.
